# The Contemporary Role of Cardiovascular Magnetic Resonance in Ischemic Cardiomyopathy

**DOI:** 10.3390/jcm14217479

**Published:** 2025-10-22

**Authors:** Desak Gede Widyawati, Lynette L. S. Teo, Ching Ching Ong, Marie Houdmont, Ping Chai, Ching-Hui Sia

**Affiliations:** 1 National University Heart Centre, National University Hospital, Singapore 119074, Singapore; 2 Department of Diagnostic Imaging, National University Hospital, Singapore 119074, Singapore

**Keywords:** ischemic heart disease, ischemic cardiomyopathy, cardiovascular magnetic resonance

## Abstract

Ischemic cardiomyopathy is the leading cause of heart failure globally. While echocardiography is the cornerstone of cardiovascular imaging, cardiovascular magnetic resonance (CMR) plays a crucial role in providing a thorough assessment of both cardiac structure and function. CMR can deliver accurate information on cardiac function, perfusion, and tissue characterization, all while avoiding ionizing radiation and overcoming challenges associated with imaging window quality. Across the different stages of ischemic heart disease, CMR provides a precise assessment of infarct age, size, and related complications, while also supporting treatment decisions and predicting recovery. This review emphasizes not only the established diagnostic and prognostic roles of CMR in ischemic cardiomyopathy but also explores emerging quantitative and artificial intelligence-based approaches poised to redefine clinical practice.

## 1. Introduction

Ischemic cardiomyopathy (ICM), characterized by systolic dysfunction of the left ventricle in the presence of significant obstructive coronary artery disease, is the leading cause of heart failure (HF) worldwide [1,2,3,4]. It is a major contributor to the rising global burden of HF, currently affecting approximately 6 million individuals and projected to reach 8 million patients by 2030 [1,4,5]. The increasing incidence has been attributed to the success of early invasive strategies implemented in acute coronary syndromes, leading to improved patient survival [4,6]. Ischemic heart disease contributes to the development of heart failure (HF) primarily through left ventricular (LV) systolic dysfunction, which may arise following an acute myocardial infarction (MI) or through a gradual, progressive decline in systolic function in the absence of overt acute coronary syndromes. Evidence from 13 multicenter HF trials has identified ICM as the underlying cause in nearly 70% of cases [1].

In the last decade, advances in non-invasive cardiovascular imaging have markedly enhanced the ability to detect, quantify, and stratify risk in both acute and chronic coronary artery disease (CAD) [7]. While echocardiography, nuclear imaging, and computed tomography remain valuable, cardiovascular magnetic resonance (CMR) has emerged as a particularly powerful modality for the evaluation of patients with known or suspected CAD [6]. Its unique strengths, including superior temporal and spatial resolution, the capacity to assess myocardial perfusion, and the absence of ionizing radiation, enable CMR to overcome key limitations of other imaging approaches [8]. CMR provides objective measurements of left ventricular (LV) structure and function, coupled with perfusion and tissue characterization capabilities [7,8]. In particular, stress perfusion CMR is accurate, safe, cost-effective, and clinically pivotal for non-invasively assessing myocardial ischemia severity and distribution. Differentiating viable yet dysfunctional myocardium from an irreversible scar is central to revascularization decision-making. In contrast, extensive scarring is strongly linked to adverse LV remodeling and increased risk of sudden cardiac death [9]. This review highlights the contemporary role of cardiovascular magnetic resonance (CMR) in ischemic cardiomyopathy and explores emerging developments that may further expand its clinical impact.

## 2. CMR Overview

CMR is a non-invasive, radiation-free modality that complements echocardiography and is regarded as the gold standard for quantifying cardiac structure and function. With ECG gating and high-temporal-resolution imaging, CMR enables precise evaluation of ventricular performance and wall motion. Although not usually a first-line test, CMR provides superior accuracy over echocardiography for assessing ventricular volumes, ejection fraction, and regional dysfunction, and is the preferred modality when acoustic windows are limited. The use of gadolinium contrast and late gadolinium enhancement (LGE) has transformed CMR into a powerful tool for myocardial tissue characterization, particularly for detecting fibrosis by differentiating ischemic (subendocardial enhancement) from nonischemic cardiomyopathies, and it helps define disease etiology. Despite its advantages, CMR still remains less accessible and more resource-intensive than echocardiography or CT. Scans require patient cooperation, such as lying flat for 30 to 60 min and for the patient to follow breath-holding instructions. It also has limited use in unstable patients. Gadolinium contrast carries the theoretical risk of nephrogenic systemic sclerosis, particularly in renal failure patients (eGFR < 15 mL/min), and image quality may be affected by cardiac devices, although wideband sequences now mitigate this in selected cases. Claustrophobia and non-MRI-compatible devices remain important practical barriers [10]. Table 1 compares the advantages and limitations of non-invasive modalities in ischemic heart disease work-up.

## 3. CMR Role for Functional Assessment

Cardiovascular magnetic resonance (CMR) provides a comprehensive functional assessment of ischemic cardiomyopathy, with cine steady-state free precession imaging serving as the gold standard for quantifying ventricular volumes, wall motion, and global function [6,15]. The superior spatial, temporal, and contrast resolution of CMR enables precise delineation of the blood–myocardium interface, yielding highly reproducible volumetric measurements that are free of geometric assumptions [8]. CMR is recommended for evaluating cardiac morphology and function in patients in whom echocardiographic evaluation is inadequate (recommendation class I C) [16]. Wall motion abnormalities reflect the severity of ischemic injury, ranging from hypokinesia in early ischemia to akinesia and, ultimately, dyskinesia with advanced myocardial damage [16]. Clinical interpretation is most often visual, based on changes in systolic wall thickening [7]. In chronic CAD, wall thinning typically reflects infarct and fibrotic remodeling, although it may also result from severe ischemia. Importantly, wall thinning does not invariably indicate nonviable myocardium. Baer et al. demonstrated that an end-diastolic wall thickness (EDWT) cutoff of 5.5 mm had 92% sensitivity but only 56% specificity for predicting recovery after revascularization [17]. Similarly, Shah et al. reported that nearly 20% of dysfunctional, thinned segments with limited scar burden remain viable, showing improved contractility and reversal of thinning following revascularization [18]. Recently, artificial intelligence (AI)-based automated or semi-automated software tools and techniques are well established to speed up the calculation of volumes, function, wall thickness, and wall motion, further improving reproducibility and allowing for a quicker reporting process [19].

## 4. Stress Perfusion CMR

Stress CMR has been recently recognized as a reliable technique for the detection of stress-inducible ischemia in suspected IHD [7,20,21]. CMR-based assessment of inducible ischemia has been shown as a cost-effective technique, with high spatial resolution to discriminate between ischemic and normal myocardium [7]. It is based on the assessment of myocardial perfusion during pharmacological stress testing with coronary vasodilators and the detection of inducible wall motion abnormalities during high-dose dobutamine infusion. The sensitivity and specificity to detect CAD ranged between 79–88% and 81–91% for dobutamine stress CMR or 67–94% and 61–85% for adenosine stress CMR, in a meta-analysis and a multicenter study. The use of a 3.0 T field scanner has been shown to provide even higher diagnostic accuracy for qualitative and quantitative analyses, and it can improve image quality and decrease dark-rim artifact [8,22,23].

### 4.1. Vasodilator Stress Perfusion

Pharmacologic vasodilators remain the cornerstone of stress perfusion CMR, with adenosine, dipyridamole, and regadenoson being the most widely used agents [6,24]. Among these, adenosine is preferred because of its short half-life and favorable safety profile. Its mechanism exploits the physiological disparity between normal and stenotic coronary arteries: adenosine induces vasodilation in normal coronary arteries, whereas flow across a severely stenotic coronary artery fails to dilate (“coronary steal” phenomenon) [7,20]. Ensuring adequate stress is essential for reliable ischemia detection. A sufficient vasodilator response is typically indicated by a modest rise in heart rate (≈10–15 beats per minute), a fall in systolic blood pressure (>10 mm Hg), and ischemic symptoms, and a splenic switch-off is helpful (absence of splenic enhancement during gadolinium infusion under adenosine administration). Lack of this physiologic response may reflect a poor vasodilator response, which helps in identifying false-negative examination results at visual assessment or false-positive results at quantitative assessment [22,25]. During peak stress after injection of the vasodilator, a gadolinium contrast-enhanced perfusion acquisition is performed [6,7,24].

Perfusion imaging with CMR relies on the differential uptake of contrast between normally perfused and ischemic or infarcted myocardium. Regions with impaired blood flow, whether due to prior infarction or inducible ischemia, demonstrate reduced contrast delivery and appear as subendocardial or transmural dark zones (perfusion defect), in contrast to the normally enhancing myocardium [7,20,24,26]. A reversible ischemic defect is defined by its presence only under stress conditions, with normal perfusion at rest and without corresponding hyperenhancement on LGE imaging. Diagnostic features of true perfusion defects include early onset during first-pass contrast arrival, persistence beyond peak myocardial enhancement for at least five cardiac cycles, and a spatial extent greater than two pixels [22,26]. On the contrary, a dark-rim artifact is a transient subendocardial low-signal during the early phases of the first-pass perfusion. Unlike true perfusion defects, these artifacts fade as myocardial enhancement progresses and lack persistence or territorial distribution [22,26]. Rest perfusion imaging, typically performed at least 10 min after stress acquisition using the same imaging planes and contrast dose without vasodilator infusion, provides an important baseline comparator [25]. Table 2 displays the stress CMR protocol at our institution.

**Figure 1:**
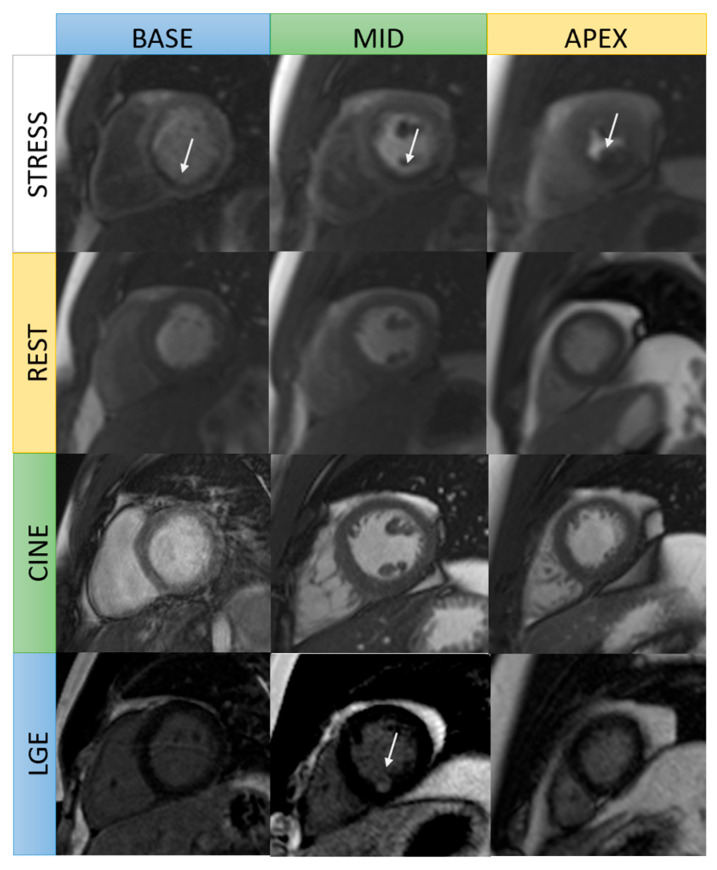
Stress CMR images in a patient with chronic total occlusion of the mid-right coronary artery. Stress and rest perfusion images: subendocardial areas at the basal to mid inferoseptal and inferior wall in stress images are in keeping with inducible ischemia (arrow). Cine images: no left ventricular wall thinning. Late gadolinium enhancement: focal subendocardial late gadolinium enhancement at the mid inferoseptal wall (arrow).

Vasodilator stress protocols have unique advantages over inotropic stressors, such as perfusion defects generally precede the development of wall motion abnormalities, and the shorter imaging protocol (3–6 min for infusion, ~1 min for image acquisition) improves both efficiency and diagnostic reliability. Comparative studies have validated these advantages, with vasodilator stress CMR demonstrating higher sensitivity for detecting CAD (93% vs. 82%) but somewhat lower specificity (66% vs. 96%) relative to dobutamine stress CMR [24]. A representative case is shown in Figure 1.

### 4.2. Inotropic Stress (Dobutamine) for Wall Motion and Contractile Reserve

Dobutamine, a sympathomimetic amine with potent inotropic and chronotropic effects, provides a pharmacologic analog to exercise by increasing myocardial oxygen demand. At low doses, it can unmask contractile reserve in hibernating myocardium—segments that appear dysfunctional at rest yet retain viability. At higher doses, dobutamine reveals ischemia in territories with hemodynamically significant coronary stenoses, expressed as inducible wall motion abnormalities in cine images [7,20]. High-dose dobutamine CMR has been shown to outperform dobutamine stress echocardiography (DSE), probably due to superior image quality and its ability to provide a better assessment of regional wall motion. However, the traditional reliance on visual wall motion interpretation introduces subjectivity and variability across observers [20]. Emerging quantitative techniques, such as strain imaging, have been implemented to overcome these limitations. Korosoglou et al. demonstrated that strain-based analysis markedly improves diagnostic accuracy for inducible ischemia during peak dobutamine stress (98% vs. 83%) [27]. Furthermore, the use of strain imaging has been shown to reduce inter-observer variability for the detection of wall motion abnormalities. Finally, blunted global longitudinal strain at stress has also been identified as an independent predictor of major adverse cardiovascular events (MACE) in patients with known or suspected coronary artery disease, with incremental value to standard clinical and imaging risk factors [7,27].

Functional cine dobutamine CMR also provides an additional option for assessing myocardial viability. At low doses (≤10 μg/kg per minute), dobutamine augments coronary vasodilation and increases myocardial contractility, allowing clinicians to distinguish viable from nonviable tissue based on the improvement of contractility in response to sympathomimetic stimulation [8,28]. Low-dose dobutamine stress CMR has demonstrated good specificity (83%) and moderate sensitivity (74%) for predicting recovery of function after revascularization [20]. Importantly, CMR-derived systolic wall thickening > 2 mm during low-dose infusion reliably identifies segments capable of functional recovery, even in previously infarcted or hibernating territories. However, this technique remains limited in clinical practice, largely because LGE imaging offers highly accurate viability assessment without the need for pharmacologic infusion or continuous monitoring [29].

Despite their utility, both vasodilator and inotropic stress techniques have important limitations, such as the risk of potential life-threatening side effects. Image acquisition is inherently time-sensitive, requiring optimal timing to capture peak stress effects. Moreover, conventional analysis of stress images relies largely on semiquantitative, visually driven interpretation (“eyeballing”), which depends on the presence of remote reference myocardium. Hence, diffuse diseases, such as coronary microvascular dysfunction, where pathological changes may be subtle, can be missed. As the clinical evidence base for stress CMR continues to expand, advances in quantitative imaging and novel acquisition strategies promise to overcome these limitations, enabling more objective, sensitive, and reproducible assessments of ischemia [7].

## 5. CMR Role in Viability

Myocardial viability reflects ischemia-induced contractile dysfunction at rest with the potential for functional recovery once normal perfusion is restored. Recognition of viable but hypocontractile myocardium is central to the management of ischemic LV dysfunction, as complete revascularization of viable tissue has been shown to improve long-term outcomes compared with medical therapy alone [12,20,24]. The concept of viability is based on the phenomena of myocardial stunning and hibernation. Stunning describes a state of reversible ventricular dysfunction persisting after brief, non-lethal ischemia despite restoration of blood flow, whereas hibernation refers to a chronically hypoperfused myocardium that receives adequate blood flow to preserve cellular metabolism but is unable to maintain contractility [16].

Assessment of myocardial viability by CMR requires a systematic approach. The evaluation begins with characterization of cardiac shape and size, wall thickness, chamber volumes, and myocardial mass. Cine CMR provides the reference standard for assessing LV contractility, where severe wall thinning, often a marker of chronic, transmural infarction, suggests nonviability. In contrast, preserved wall thickness in diastole in regions of known infarction may indicate viable myocardium. However, measurement of myocardial thickness is important but may be insufficient in predicting viability. In a large observational study, Shah et al. demonstrated that even markedly thinned segments (<5.5 mm) without evidence of scarring on LGE retained the potential for functional recovery after revascularization [18,29].

**Figure 2:**
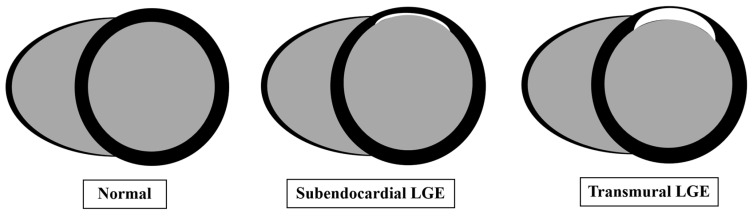
Comparison of late gadolinium enhancement patterns in normal heart and ischemic heart disease.

Since its initial application, LGE has become the gold standard for assessing myocardial viability in ischemic heart disease [18,29]. Beyond its role in viability testing, LGE should be performed to differentiate between ischemic and nonischemic cardiomyopathies and as a powerful predictor of recovery following revascularization [15,16]. The strength of contrast-enhanced CMR lies in its ability to detect subendocardial infarction and define the transmural extension of myocardial injury, with high spatial resolution (1–3 mm in-plane) and excellent signal-to-noise ratios [9]. The technique uses the kinetics of gadolinium-chelate contrast agents, which rapidly wash out of healthy myocardium but persist in necrotic or fibrotic regions due to extracellular expansion [15]. Inversion recovery preparation pulse sequences are used in LGE imaging to suppress the normal myocardial signal, allowing areas of necrosis or replacement fibrosis to appear as regions of hyperenhancement (Figure 2). This approach enables the detection of even small myocardial infarcts (≥0.7 g of myocardial mass) [8].

Various LGE patterns can be used to differentiate between ischemic and nonischemic cardiomyopathies [30]. In ischemic heart disease, contrast uptake occurs along the ischemic wavefront from subendocardium to epicardium with varying degrees of transmurality, usually affecting one or more coronary territories (Figure 1) [6,30]. Transmural infarction extends through the entire wall of myocardium; non-transmural infarction has a sub-endocardial origin and involves <100% of wall thickness [30]. Non-ischemic cardiomyopathies, on the other hand, show mid-wall, sub-epicardial, or mixed LGE pattern, and are not confined to one coronary artery territory [31]. However, mid-wall LGE, a typical finding in non-ischemic cardiomyopathy, may also be present in an estimated 10% of patients with ischemic cardiomyopathy. This is indicative of extensive remodeling with severe LV dilation and systolic dysfunction and is strongly associated with ventricular arrhythmias and increased all-cause mortality, particularly when extensive or localized within the septum or lateral wall [32,33].

**Figure 3:**
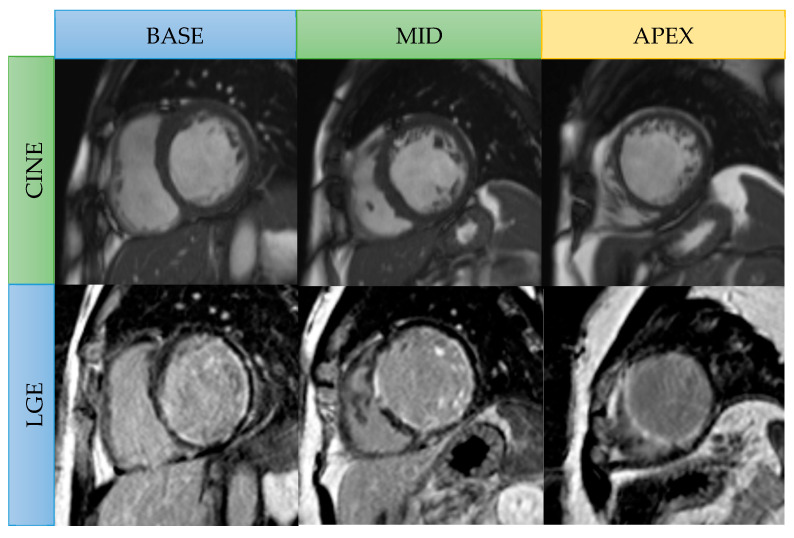
Viability CMR images in a 56-year-old male patient with three-vessel CAD. Cine SSFP short-axis images: Focal thinning of the myocardium at the mid cavity anteroseptal and anterior wall, apical septal, apical anterior, and apical inferior wall. Late gadolinium enhancement (LGE): Transmural LGE in the mid anteroseptal wall, apical septal, and apical inferior left ventricular walls (left anterior descending coronary artery territory) represent nonviable segments. Subendocardial LGE presents in the basal-to-mid inferolateral wall (left circumflex artery territory) and in the mid inferoseptal wall (right coronary artery territory), representing hibernating segments (viable).

Myocardial scars carry prognostic significance and reflect irreversible injury; however, not all thinned myocardia are associated with nonviable tissue. Regions with an end-diastolic wall thickness ≤5.5 mm may still demonstrate recovery of contractility and wall thickening after revascularization if the scar burden is limited (≤50% transmural extent of infarction) [11]. Akinetic segments with minimal or no subendocardial scars have >90% likelihood of functional recovery when revascularization is successful [19]. The transmural extent of LGE remains the most powerful predictor of post-revascularization outcomes: segments with >50% transmural scar have <10% probability of functional recovery, whereas those with less extensive involvement frequently regain contractility [6,9]. A meta-analysis by Romero et al. confirmed that an LGE threshold of <50% transmurality offers high sensitivity and strong negative predictive value for predicting recovery [8,34]. Similarly, Kim et al. demonstrated that functional recovery was 78% in segments without scarring, but in <2% of those with scar transmurality > 75% [8,29]. A representative case is shown in Figure 3.

Conventional bright-blood LGE can be challenging in detecting small subendocardial infarcts, as the signal intensity of enhancing myocardium may be difficult to distinguish from the adjacent blood pool, particularly in the early post-contrast injection, which may lead to missed subtle subendocardial involvement [8,16]. To address this limitation, emerging black-blood (dark-blood) LGE techniques have been developed, offering superior contrast between blood and myocardium and thereby improving detection of subendocardial MI [9].

Currently, no universally accepted standard exists for quantifying LGE. Multiple techniques have been proposed, ranging from manual contouring to semi-automated signal-intensity-based thresholds (2–6 SD above normal myocardium), the full-width-at-half-maximum (FWHM) technique, and nonbinary methods. Most studies define core infarct as regions ≥ 5 SD above normal myocardium and the peri-infarct ‘border zone’ as 2–3 SD above normal myocardium, though the full width at half maximum technique (FWHM) approach has consistently demonstrated superior accuracy and reproducibility across myocardial diseases [20]. Despite these advances, current techniques remain time-consuming, require the definition of regions of interest in normal and/or enhanced myocardium, and yield variable results across methods [7].

## 6. CMR Role in Prognostication

CAD is the major cause of sudden cardiac death, and myocardial scarring provides the arrhythmogenic substrate for re-entrant VT. Patients with previous myocardial infarction and left ventricular systolic dysfunction (LVSD) are especially at risk of potentially life-threatening ventricular arrhythmias [35]. As such, an accurate measurement of infarct scarring is a key part of risk stratification, as scarring also represents the anatomic substrate for malignant re-entrant circuits, propagating sudden cardiac death following myocardial infarction [21]. In ischemic cardiomyopathy, higher scar burden has also been shown to be an independent predictor of mortality or heart transplantation; therefore, confirming its value in terms of prognosis beyond common parameters (e.g., LVEF) [9]. Research on patients undergoing CMR prior to implantable cardioverter defibrillator (ICD) placement shows that scar quantification is a more powerful predictor of arrhythmic events, such as sudden cardiac death, appropriate ICD shocks, and inducibility at EP study, than LVEF. This prognostic value is also maintained among patients with preserved systolic function and demonstrates the distinctive contribution of CMR scar characterization for arrhythmic risk evaluation; CMR is thus also a valuable tool for the assessment of patients prior to ICD implantation, and there is recent evidence that scar extent on CMR predicts the response to cardiac resynchronization therapy (CRT). In a study on 47 IHD patients undergoing CRT, CMR showed that response rate to CRT was higher in patients with higher LVEF, smaller scars, and a lower number of LV segments with >51% scar transmurality [21].

Furthermore, in a multicenter registry from 13 US centers, Ge et al. [36] reported that in patients with reduced LVEF (<50%) referred for stress CMR for suspected myocardial ischemia, an annual primary outcome event rate of 1.1% was reported in patients with no CMR evidence of ischemia or LGE, and higher event rates were associated with the presence of ischemia and/or LGE. At median follow-up of 5.0 years, ischemia and LGE were independent predictors of the primary (hazard ratio [HR]: 2.63; 95% confidence interval [CI]: 1.68 to 4.14; *p* < 0.001; and HR: 1.86; 95% CI: 1.05 to 3.29; *p* = 0.03) and secondary (HR: 2.14; 95% CI: 1.55 to 2.95; *p* < 0.001; and HR 1.70; 95% CI: 1.16 to 2.49; *p* = 0.007) outcomes [36].

## 7. Recent Trials and Clinical CMR Application in Guiding Revascularization

The question of whether revascularization improves outcomes in ischemic cardiomyopathy remains a defining debate in contemporary care [20]. The Surgical Treatment for Ischemic Heart Failure (STICH) trial provided pivotal evidence that CABG plus optimal medical therapy (OMT) confers a durable survival advantage over OMT alone in patients with coronary artery disease and severe LV dysfunction (LVEF ≤ 35%), with sustained reductions in all-cause mortality and heart failure hospitalizations at 10 years. Strikingly, this benefit was independent of myocardial viability, overturning the traditional dogma that revascularization decisions should hinge on viability testing [20,28]. In contrast, the Revascularization for Ischemic Ventricular Dysfunction-British Cardiovascular Intervention Society-2 (REVIVED-BCIS2) trial showed that PCI, even in patients with viable myocardium, failed to improve survival, LV function, or heart failure outcomes when added to contemporary OMT [36,37,38]. Subsequent pooled analyses reinforced a consistent message: CABG, not PCI, remains the only revascularization strategy with proven long-term survival benefit in ischemic LV dysfunction [28,39].

Myocardial viability testing aims to identify patients with ischemic cardiomyopathy who may derive prognostic or functional benefit from revascularization. These assessments classify myocardial tissue into three categories: normally contracting viable myocardium, dysfunctional but viable (“hibernating”) myocardium with potential for functional recovery, and nonviable scar tissue in which contractile improvement is unlikely [40]. However, current evidence suggests that viability or ischemia testing is not mandatory for guiding revascularization decisions. Nonetheless, documenting myocardial viability, optimally assessed with contemporary CMR or PET, remains a rational and clinically intuitive step, particularly when surgical risk complicates the decision-making process. While ischemia testing has less consistent impact on management, especially in asymptomatic patients, emerging retrospective data in broader populations indicate that ischemia-guided revascularization may offer benefit when the ischemic burden is extensive (>15% of the left ventricular myocardium) [39].

## 8. Future Directions

Late gadolinium enhancement is a strong predictor for primary and secondary events in ischemic cardiomyopathy. However, no universally accepted standard exists for quantifying LGE. Precise and fully automated LGE quantification remains largely investigational, with important promise for research but without widespread clinical implementation [7].

Although visual interpretation remains the cornerstone of clinical stress perfusion CMR, quantitative perfusion (qPerf) CMR represents the next promising technique by enabling full quantification of myocardial blood flow (MBF). qPerf CMR improves diagnostic accuracy for ischemia detection and ischemic burden assessment. It allows the conversion of dynamic MRI signal intensity to change over time into gadolinium-based contrast agent concentrations to provide stress and rest myocardial blood flow (MBF) measurements (in mL/min/g). At rest, MBF remains constant over a wide range of perfusion pressures due to coronary vascular resistance autoregulation, while under stress conditions the MBF can increase up to four- to five-fold to match to the increased oxygen demand. The most significant added value of qPerf CMR is its greater capability in the distinction of single-vessel from multi-vessel disease and in the diagnosis of microvascular dysfunction. Given the relative novelty of qPerf CMR, further research in larger cohorts and across a broader spectrum of diseases can be used to define the role of this examination in clinical practice [22,41].

## 9. Conclusions and Future Outlook

CMR is a highly accurate, robust, and non-invasive modality that allows multi-parametric assessment of ischemic heart disease. In ischemic cardiomyopathy, it provides unparalleled assessment of ventricular function, scar burden, and myocardial viability, directly informing prognosis and guiding therapeutic decision-making. However, many conventional techniques are unable to provide accurate automated assessments of tissue properties (such as myocardial blood flow and the degree and extent of tissue viability). As a result, new quantitative techniques like parametric mapping, strain imaging, quantitative perfusion, as well as artificial intelligence-powered analysis, are emerging to be incorporated into clinical practice, which could enhance the diagnostic and prognostic capabilities of CMR in the future.

## 10. Take-Home Messages

- Contemporary CMR has evolved from a diagnostic adjunct to a central tool in the the phenotyping and prognostication of ischemic cardiomyopathy.
- Quantitative mapping and automated perfusion analyses now bridge the gap between structural and functional assessment, offering reproducible metrics that could reshape clinical decision-making.
- While the prognostic value of LGE is established, its integration with artificial intelligence and stress perfusion imaging offers opportunities for personalized risk stratification.
- The divergence between STICH and REVIVED trials highlights the need to redefine the role of CMR-derived viability beyond revascularization candidacy, toward comprehensive myocardial health assessment. This requires further research.
- Future work should focus on standardizing quantitative techniques and integrating CMR with multimodal imaging and molecular markers to refine management of ischemic LV dysfunction.

## Figures and Tables

**Table 1:** Comparison of mon-invasive multimodality imaging in ischemic heart disease work-up. References [11,12,13,14].

Modality	Advantages	Limitations
Echocardiography	Low cost Widely accessible Good safety profile Portable options available (allows for bedside evaluation) Provides crucial information on cardiac anatomy, systolic and diastolic function, regional wall motion abnormalities, and associated valvular heart disease Exclusion of alternative causes of chest pain: pericarditis, pulmonary embolism, aortic dissection, hypertrophic cardiomyopathy	Operator dependence Limited by poor acoustic window LVEF relies on geometric assumptions (dependent on load, heart rate, and influenced by changes in LV geometry)
Stress echocardiography	Assessment of known or suspected CAD with high diagnostic and prognostic value Low cost More widely available Avoidance of ionizing radiation Able to detect myocardial ischemia by identification of wall motion abnormalities Several prognostic parameters, including blood exercise duration, pressure response, ST-T changes, and LV dilatation, can be simultaneously assessed and enhance their diagnostic and prognostic role Very high negative predictive value (98.8%) for primary and secondary events	Operator dependence Limited by poor acoustic window Contraindicated in uncontrolled hypertension, severe arrhythmias, significant left ventricular outflow tract obstruction, ACS, and symptomatic severe aortic stenosis
CCTA	Main non-invasive imaging test to detect epicardial coronary atherosclerotic plaque, severity of stenosis, and segments involved Enable analysis of coronary plaque composition (well-known association of high-risk plaque and increased incidence of acute coronary syndrome) High negative predictive value for CAD evaluation Qualitative and quantitative assessment of coronary stenosis (CAD-RADS score) and risk stratification Can identify some noncardiac causes of chest pain	Radiation exposure Contrast injection Low positive predictive value for CAD evaluation Limited role for evaluation of hemodynamically significant CAD Potentially reduced quality in patients with morbid obesity, high or irregular heart rates, or severe coronary calcification (calcium blooming artifacts)
Fractional flow reserve (FFRct)	Increase positive predictive value for depiction of significant CAD Allows functional assessment of the plaque over the anatomical coronary stenosis Allows reduction in invasive coronary angiography	Require high quality CT data Limited availability Long processing time
Stress CT perfusion	For evaluation of myocardial ischemia over the plaque stenosis Increased diagnostic accuracy over CCTA when stress CT perfusion is applied Higher prognostic value for prediction of MACE compared to CCTA or FFRct	High radiation exposure Needs the administration of a contrast agent
Stress CMR	Non-invasive Can assess wall motion, ischemia, and infarction in one study. Can quantify myocardial blood flow to improve test accuracy and assess myocardial and pericardial diseases Can evaluate viability and presence of inducible ischemia Significant prognostic implications: a negative test is associated with a very low incidence of cardiac events, a low need for coronary revascularization, low cost of subsequent ischemia testing Reduce the risk of unnecessary invasive coronary angiography Lack of ionizing radiation Allow tissue characterization with late gadolinium enhancement imaging to differentiate between ischemic and non-ischemic cardiomyopathy and viability assessment	Low availability Require high level of expertise More costly Need of contrast and stress agents. Need patient cooperation to lie flat and follow breath-holding instructions Limited used in severe renal failure patients, patients with non-MRI compatible devices and claustrophobia patients Time-consuming nature
Single-photon emission computed tomography (SPECT)	Functional modality for assessing the hemodynamic significance of coronary lesion and viability Extensive clinical experience and abundance of studies demonstrating the ability of SPECT in diagnosis, risk stratification, treatment, and prognostic evaluation Can identify high-risk patients who might have reduced mortality with early revascularization compared to medical therapy Can be performed in all patients	Underestimating the true extent of obstructive epicardial coronary disease (e.g., balanced ischemia) Inability to determine microvascular dysfunction Exposure to radiation Attenuation, motion, and soft tissue artifacts may underestimate extent of diseases. Lower sensitivity and specificity when compared with MRI, PET, and CT
Myocardial Positron Emission Tomography (PET) imaging	Clinical reference standard for the quantification of myocardial perfusion Offers higher sensitivity and spatial resolution Ability to assess contractile function abnormalities with gated imaging Quantify global and regional myocardial blood flow (MBF) May detect microvascular diseases Can be performed in patients with arrhythmias, advanced kidney disease, and pacemakers and/or defibrillators Assess myocardial function and metabolism	More expensive Radiation exposure Limited availability

**Table 2:** Cardiac MRI protocol for stress perfusion at our institution.

Step	Sequences	Acquisition Planes	Primary Goals
Anatomic survey	Bright blood and dark blood single-shot imaging	Axial, sagittal, coronal stack	Assessment of cardiovascular anatomy, planning of planes, and evaluation of extracardiac findings
Cine imaging	Balanced steady-state free precession imaging	Long axis-single slice: 2 chamber, 3 chamber, 4 chamber view	Assessment of cardiac anatomy and function
Stress perfusion (with adenosine infusion)	Real-time cine	3 slices short axis: basal, mid, apical LV	Detection of inducible ischemia
Cine imaging	Balanced steady-state free precession imaging	Short-axis stack	Assessment of cardiac anatomy and 3D volume, and function
Rest perfusion	Real-time cine	3 slices short axis: basal, mid, apical LV	Comparison to stress perfusion imaging
Late gadolinium enhancement (LGE)	LGE imaging: magnitude and phase sensitive inversion recovery	Short-axis stack Long axis-single slice: 2 chamber, 3 chamber, 4 chamber view	Detection of infarction or fibrosis

## Data Availability

Not applicable.
